# Cyclobenzaprine-related adverse events: a comprehensive pharmacovigilance analysis using the FDA Adverse Event Reporting System

**DOI:** 10.3389/fmed.2025.1574395

**Published:** 2025-09-22

**Authors:** Jiakuan Tu, Chaoxiang Zhang, Shuihua Xie, Jianhua He, Hao Zhang

**Affiliations:** ^1^The First Department of Orthopedics, Jiangxi Provincial Hospital of Integrated Traditional Chinese and Western Medicine, Nanchang, China; ^2^Jiangxi University of Traditional Chinese Medicine, Nanchang, China

**Keywords:** cyclobenzaprine, adverse events, pharmacovigilance, FAERS, musculoskeletal pain, safety signals

## Abstract

**Background:**

Cyclobenzaprine, a centrally-acting muscle relaxant, is widely used for the treatment of musculoskeletal pain. Despite its efficacy, concerns regarding its safety profile, particularly adverse events (AEs), have been increasingly reported. This study aims to comprehensively analyze cyclobenzaprine-related AEs using the FDA Adverse Event Reporting System (FAERS) database to identify potential safety signals and inform clinical practice.

**Methods:**

A retrospective pharmacovigilance study was conducted using FAERS data from Q1 2004 to Q3 2024. Reports involving cyclobenzaprine as the primary suspect drug were analyzed. Descriptive statistics and disproportionality analyzes, including Reporting Odds Ratio (ROR), Proportional Reporting Ratio (PRR), Bayesian Confidence Propagation Neural Network (BCPNN), and Multinomial Gamma Poisson Shrinkage (MGPS), were employed to detect safety signals. Subgroup analyzes were performed to explore demographic heterogeneity. Furthermore, the Weibull distribution was utilized to construct a model for the risk of adverse events as time progresses.

**Results:**

Out of 18,289,374 AE reports, 2,425 were linked to cyclobenzaprine. Employing four distinct computational approaches at the preferred term (PT) level, we pinpointed 1,100 PTs signifying remarkable adverse reactions. The adverse reactions listed on the drug’s label, like cardiac arrest, respiratory arrest, drug hypersensitivity, dizziness, and somnolence, presented conspicuous signals. In addition, we discovered potential adverse reactions not detailed on the label, for example, toxicity to various agents, completed suicide, drug abuse, overdose, drug interaction, and confusional state. Subgroup analysis brought to light gender—specific AEs. Males had a higher likelihood of experiencing delirium and hallucinations, whereas females were more inclined to encounter drug hypersensitivity and muscle spasms. The vast majority of these AEs were reported during the first month of cyclobenzaprine treatment, with a median onset time of 7 days.

**Conclusion:**

This study confirms known AEs associated with cyclobenzaprine and identifies new potential risks, such as toxicity and suicidal behavior. These findings underscore the need for enhanced monitoring and further research to mitigate risks, particularly in vulnerable populations. Clinicians should remain vigilant for both somatic and psychiatric AEs when prescribing cyclobenzaprine, especially in patients with a history of mental health issues or substance abuse.

## 1 Introduction

Pain is one of the most common symptoms in acute conditions, and in half of the cases involving pain, the final diagnosis is due to musculoskeletal (MSK) causes (1). MSK pain, which includes a variety of conditions, such as lumbar, cervical, and thoracic pain significantly impacts global health (2). Recent data indicate that one in three to four adults over the age of 18 experiences MSK pain, with a prevalence comparable to the combined incidence of cardiovascular and chronic respiratory diseases (3). MSK pain can stem from various disorders, including inflammation and neuropathy (4), and is often exacerbated by abnormalities in muscle tone (5). MSK-associated pain and inflammatory pain are distinct yet overlapping clinical entities (4). MSK-associated pain primarily arises from structural or functional impairments of musculoskeletal tissues, typically presenting as activity-exacerbated pain with localized tenderness and limited range of motion (6). In contrast, inflammatory pain is driven by pro-inflammatory mediators (e.g., cytokines, prostaglandins) released during tissue inflammation, characterized by persistent pain at rest, erythema, swelling, and elevated inflammatory biomarkers (7). And in 2011, it cost 213 billion United States dollars, equivalent to 1.4% of the gross domestic product (8). In clinical practice, for the patient population suffering from MSK pain, the muscle relaxant cyclobenzaprine is a widely used treatment option.

Cyclobenzaprine, a centrally-acting muscle relaxant, functions as a serotonin receptor antagonist. It reduces muscle tone by inhibiting serotonergic descending pathways in the spinal cord (9, 10). This mechanism makes it independently indicated for non-inflammatory MSK pain associated with muscle spasms—in such cases, it targets the underlying muscle hypertonia without requiring concurrent anti-inflammatory agents (11). For inflammatory MSK pain, cyclobenzaprine is often used adjunctively with anti-inflammatory drugs to address muscle spasm superimposed on inflammatory processes; however, it is not independently indicated for pure inflammatory pain, as it lacks direct anti-inflammatory activity (12). Notably, its role varies by pain chronicity: in acute MSK pain, it acts via spinal serotonergic inhibition to reduce muscle hypertonia, consistent with its short-term (≤2 weeks) FDA indication (13, 14). For chronic pain (>3 months), it is not first-line—chronic pain often involves central sensitization or inflammation, which cyclobenzaprine does not target, and long-term use raises AE risks, aligning with guidelines cautioning against prolonged use (15, 16).

Numerous clinical trials have confirmed that cyclobenzaprine can achieve improvement in MSK pain (17), for example, David G Borenstein et al. found that a low-dose regimen of cyclobenzaprine hydrochloride for acute skeletal muscle spasms significantly increased the average efficacy scores (18). However, with the increasing use of cyclobenzaprine in clinical practice, adverse reactions have also increased. Adverse reactions often occur on the labels, including digestive issues like vomiting, stomach pain, and bloating; cardiovascular problems such as tachycardia, arrhythmia, and hypotension; and common symptoms like dizziness, drowsiness, and local muscle weakness (12, 19, 20). Thus, clinicians treating MSK pain with cyclobenzaprine should be vigilant about potential adverse reactions to manage them promptly.

The FDA Adverse Event Reporting System (FAERS) aggregates voluntary adverse event (AE) reports submitted by consumers, physicians, and pharmacists (21). It is a crucial resource for evaluating the real-world safety of medications (22). Recently, numerous pharmacovigilance studies using the FAERS database have been published, underscoring its recognized reliability for assessing drug safety profiles (23, 24). Thus, we aim to extract data from the FAERS database on cyclobenzaprine’s clinical use for MSK pain to better serve patients.

## 2 Methods

### 2.1 Data source and collection

**Figure 1:**
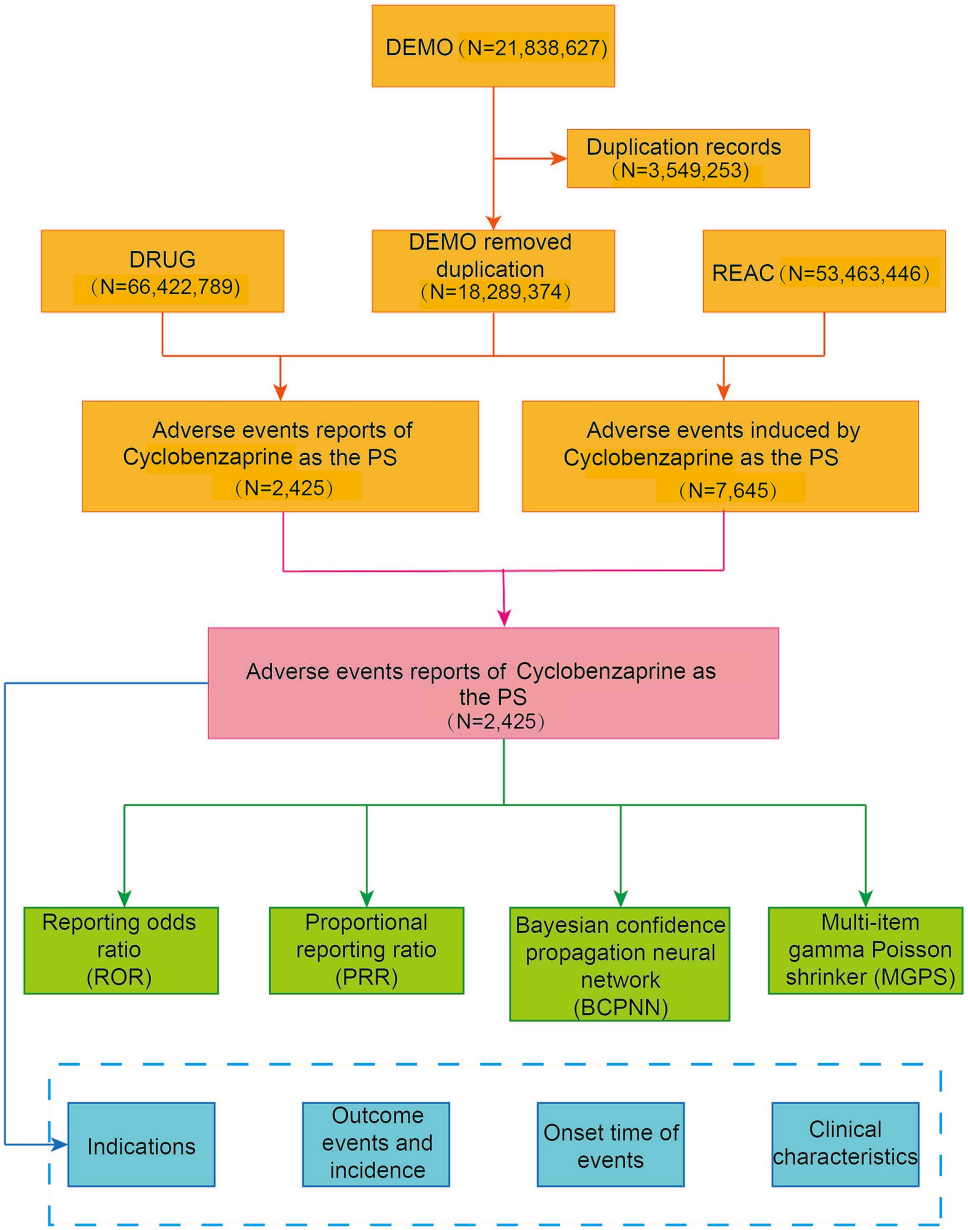
Flowchart demonstrating the adverse event analysis process for cyclobenzaprine using the FAERS database.

Cyclobenzaprine was approved by the FDA in 1977; however, the FAERS database, which we utilized for this retrospective pharmacovigilance assessment, only contains records from Q1 2004 onwards. Our analysis therefore focused on AE reports associated with cyclobenzaprine between Q1 2004 and Q3 2024. The FAERS database amasses data from diverse sources. These sources consist of demographic and administrative particulars (DEMO), adverse drug reactions (REAC), patient outcomes (OUTC), drug—specific details (DRUG), therapy timelines (THER), reporting entity details (RPSR), and indications for use (INDI) (25). This data was utilized to classify AEs in connection with the drug exposures of individual patients. AEs were encoded by means of the Medical Dictionary for Regulatory Activities (MedDRA) preferred term (PT) codes. These codes are structured within a hierarchical system that encompasses system organ class (SOC), high-level group term (HLGT), and high-level term (HLT) (26, 27). For the purpose of guaranteeing analytical accuracy, within the DEMO table, we eliminated duplicate reports. This was achieved by keeping the entry with the most recent FDA_DT for matching CASEID and FDA_DT (28). When no match was found, we chose the record with the higher PRIMARYID (29). From the FAERS database, we retrieved a total of 18,289,374 AE reports. Among these, 2,425 reports identified cyclobenzaprine as the primary suspect (PS) drug. The methodology of our study is presented in a detailed flowchart, as shown in Figure 1.

### 2.2 Statistical analysis

Descriptive statistical methods were employed to characterize the profiles of AE reports linked to cyclobenzaprine. To assess the strength of the association between the drug and specific AEs, disproportionality analysis was performed by comparing observed frequency ratios in drug-exposed and non-exposed groups through a four-cell table (Supplementary Table 1). This approach was applied within the framework of our study to detect potential safety signals. The reporting odds ratio (ROR), proportional reporting ratio (PRR), Bayesian Confidence Propagation Neural Network (BCPNN), and Multinomial Gamma Poisson Shrinkage (MGPS) were utilized to validate an elevated risk of AEs associated with cyclobenzaprine. An AE was classified as a potential adverse drug reaction if it surpassed the predefined positivity threshold in any of these methods (30). Notably, higher values derived from these metrics indicate a stronger signal and a more significant association between the suspected drug and the AE. Detailed methodologies and thresholds for these disproportionality analyzes are outlined in Supplementary Table 2. All statistical analyzes were conducted using R software, version 4.2.2.

## 3 Results

### 3.1 Descriptive analysis

**Figure 2:**
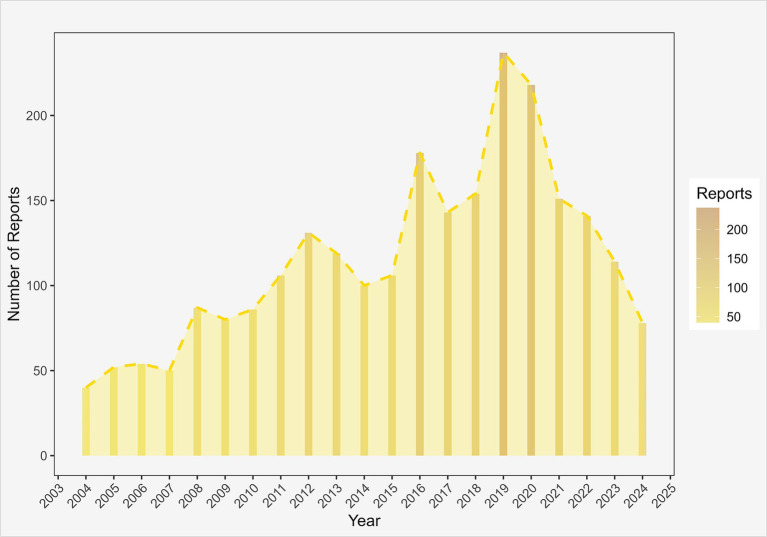
Number of AEs reported annually since cyclobenzaprine was introduced to the market.

A total of 18,289,374 AE reports were extracted from the FAERS database between Q1 2004 and Q3 2024 for analysis. Among these reports, cyclobenzaprine was recorded as the primary suspect drug in 2,425 cases, indicating a potential association with the reported AEs. Between 2004 and 2019, the number of AE reports associated with cyclobenzaprine showed an overall upward trend, with minor fluctuations observed during this period. The highest number of cases was recorded in 2019, with a total of 237 reports. However, starting from 2020, a gradual decline in the number of AE reports was noted (Figure 2). This surge in AE reports during 2019–2020 may be primarily attributed to the COVID-19 pandemic: widespread lockdowns led to increased musculoskeletal pain (e.g., muscle spasms and back pain) due to prolonged sedentary behavior and poor ergonomics, expanding cyclobenzaprine prescribing volumes, while enhanced clinical monitoring of drug–drug interactions and elevated public health awareness further promoted AE reporting (31–33). The subsequent decline from 2021 onward likely reflects the resumption of pre-pandemic lifestyles for reducing new musculoskeletal pain cases, optimized prescribing strategies by prioritizing short-term use and alternative muscle relaxants, and a return to rational AE reporting practices as pandemic-related health anxiety diminished (34).

Clinical characteristics of cyclobenzaprine-related reports from the FAERS database are presented in Table 1. Analysis of the data revealed a higher likelihood of AEs in females (53.8%) than in males (31.3%). Additionally, age was moderately associated with AE prevalence, with the highest rates observed in the 18 to 65 age group (52.5%), followed by the 65 to 85 age group (9.9%). In contrast, lower prevalence rates were noted in individuals under 18 years old (3.1%) and those over 85 years old (0.9%). Geographically, the majority of AE reports originated from the United States, constituting 85.4% of the total, while Canada contributed 9.3%. Reports from all other countries combined accounted for less than 5.3%. Notably, the distribution of reporters showed that consumers accounted for 27.7% of AE reports, followed by medical doctors (27.6%), pharmacists (11.6%), and other health professionals (15.8%), with 7.6% of reports from unspecified sources. The most frequently reported indication was Muscle Spasms (8.5%), followed by Back Pain (5.9%), Pain (4.4%), Suicide Attempt (3.1%), and Muscle Relaxant Therapy (2.5%). These indications align with those approved by the FDA.

**Table 1:** Clinical characteristics of reports with cyclobenzaprine from the FAERS database.

Characteristics	Case number	Proportion (%)
Number of events	2,425	
Gender (%)		
Female	1,305	53.8
Male	760	31.3
Not specified	360	14.8
Weight (kg)		
<50	21	0.9
>100	98	4.0
50 ∼ 100	514	21.2
Not specified	1792	73.9
Age (years)		
<18	74	3.1
18 ∼ 65	1,273	52.5
65 ∼ 85	241	9.9
>85	21	0.9
Not specified	816	33.6
Reported countries		
United States	2070	85.4
Canada	225	9.3
Portugal	17	0.7
Spain	10	0.4
Germany	8	0.3
Others	95	3.9
Reporter		
Consumer	672	27.7
Medical Doctor	669	27.6
Other health-professional	384	15.8
Health professional	231	9.5
Pharmacist	281	11.6
Lawyer	4	0.2
Not specified	184	7.6
Year of report		
2004	40	1.6
2005	52	2.1
2006	54	2.2
2007	50	2.1
2008	87	3.6
2009	80	3.3
2010	86	3.6
2011	106	4.4
2012	131	5.4
2013	119	4.9
2014	100	4.1
2015	106	4.4
2016	178	7.3
2017	143	5.9
2018	154	6.4
2019	237	9.8
2020	218	9.0
2021	151	6.2
2022	141	5.8
2023	114	4.7
2024	78	3.2
Outcomes		
Death	824	34.0
Other serious outcome	421	17.4
Hospitalization	361	14.9
Life-Threatening	108	4.5
Disability	41	1.7
Required intervention to prevent permanent impairment	16	0.7
Cancer	13	0.5
Not specified	641	26.4
Indications		
Muscle spasms	205	8.5
Back Pain	142	5.9
Pain	107	4.4
Suicide attempt	76	3.1
Muscle relaxant therapy	61	2.5
Not specified	1834	75.6

### 3.2 Time to event onset

Between the first quarter of 2004 and the third quarter of 2024, our study gathered 281 reports that detailed the timing of AEs. The median duration until the onset of an AE was 7 days, and the interquartile range spanned from 3 to 45 days.

**Figure 3:**
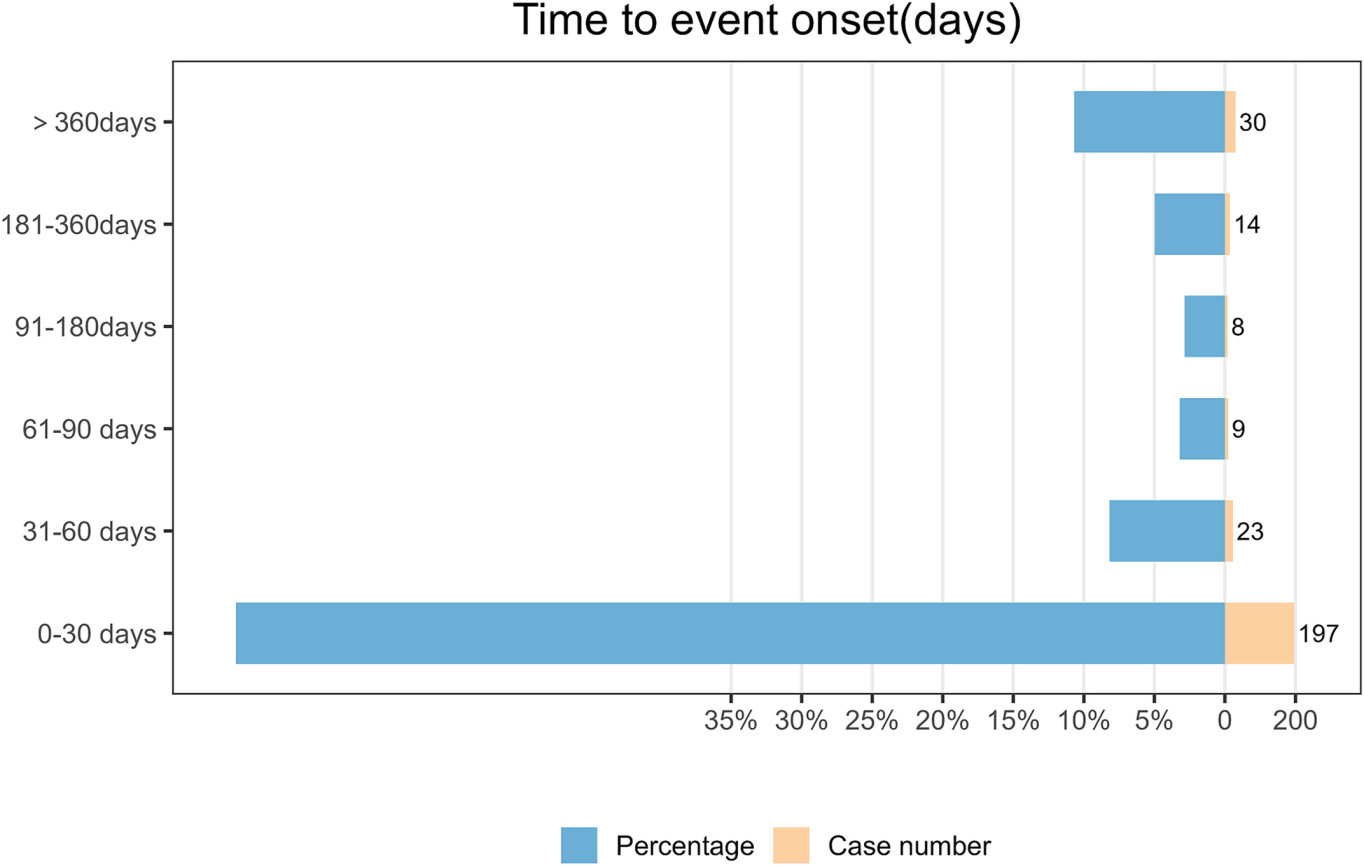
Time to event onset.

As depicted in Figure 3, the largest number of AEs related to cyclobenzaprine took place within the initial 30 days of treatment (*n* = 197, 70.1%). Conversely, the AEs reported during the 31–60-day period (*n* = 23, 8.2%), 61–90-day period (*n* = 9, 3.2%), 91–180-day period (*n* = 8, 2.8%), 181–360-day period (*n* = 14, 5.0%), and after 360 days of treatment (*n* = 30, 10.7%) were noted at substantially lower percentages. The analysis of the Weibull Shape Parameter (presented in Table 2) uncovered an estimated shape parameter (*β*) of 0.38, with a 95% confidence interval (CI) fluctuating between 0.35 and 0.41. This *β* value signified a downward trend in the occurrence of AEs as time elapsed, indicating a pattern frequently associated with early-stage adverse reactions.

**Table 2:** Time to onset of cyclobenzaprine-associated adverse events and Weibull distribution analysis.

Drug	Time to onset (days)		Weibull distribution		
	Case reports	Median (IQR)	Scale parameter: *α* (95% CI)	Shape parameter: *β* (95%CI)	Type
Cyclobenzaprine	281	7 (3–45)	46.90 (31.44–62.36)	0.38 (0.35–0.41)	Early failure

CI, confidence interval; IQR, interquartile range.

### 3.3 System organ class level of AEs distribution

**Figure 4:**
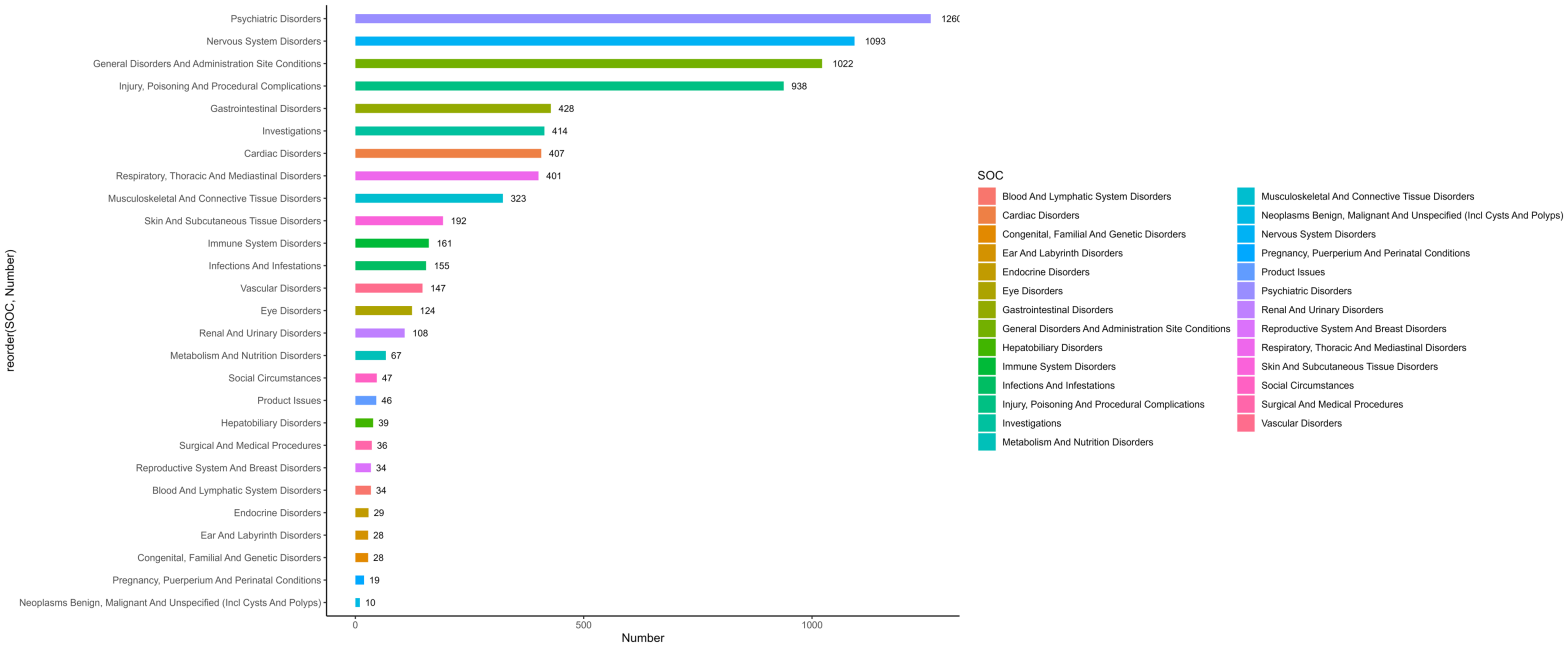
The number of AEs related to cyclobenzaprine at the SOC level in the FAERS database.

The AEs associated with cyclobenzaprine affected 26 of the 27 SOCs. A comprehensive analysis of significant signals within these SOCs is detailed in Table 3. Key findings included psychiatric disorders (*n* = 1,260, ROR 3.25 [3.06–3.45], PRR 2.88 [1639.47], EBGM 2.88 [2.74], IC 1.53 [−0.14]), nervous system disorders (*n* = 1,093, ROR 1.79 [1.68–1.91], PRR 1.68 [328.2], EBGM 1.68 [1.59], IC 0.75 [−0.92]), injury, poisoning, and procedural complications (*n* = 938, ROR 1.35 [1.26–1.45], PRR 1.31 [75.36], EBGM 1.31 [1.24], IC 0.39 [−1.28]), cardiac disorders (*n* = 407, ROR 2.05 [1.86–2.27], PRR 2 [208.64], EBGM 2 [1.84], IC 1 [−0.67]), immune system disorders (*n* = 161, ROR 1.91 [1.63–2.23], PRR 1.89 [68.34], EBGM 1.89 [1.66], IC 0.92 [−0.75]), social circumstances (*n* = 47, ROR 1.42 [1.06–1.89], PRR 1.41 [5.7], EBGM 1.41 [1.11], IC 0.92 [−0.75]), and endocrine disorders (*n* = 29, ROR 1.49 [1.04–2.15], PRR 1.49 [4.66], EBGM 1.49 [1.1], IC 0.57 [−1.09]). These results highlight the primary organ systems most frequently affected by AEs during cyclobenzaprine treatment, emphasizing the need for enhanced surveillance and further research to better understand and mitigate these risks. The distribution of AEs at the SOC level is visually represented in Figure 4.

**Table 3:** The signal strength of AEs related to cyclobenzaprine at the SOC level in the FAERS database was detected by four algorithms.

System organ class (SOC)	Case reports	ROR (95% CI)	PRR (95% CI)	EBGM (EBGM05)	IC (IC025)
Psychiatric disorders*	1,260	3.25 (3.06–3.45)	2.88 (1639.47)	2.88 (2.74)	1.53 (−0.14)
Nervous system disorders*	1,093	1.79 (1.68–1.91)	1.68 (328.2)	1.68 (1.59)	0.75 (−0.92)
General disorders and administration site conditions	1,022	0.73 (0.68–0.78)	0.77 (89.16)	0.77 (0.72)	−0.39 (−2.05)
Injury, poisoning and procedural complications*	938	1.35 (1.26–1.45)	1.31 (75.36)	1.31 (1.24)	0.39 (−1.28)
Gastrointestinal disorders	428	0.63 (0.57–0.7)	0.65 (86.52)	0.65 (0.6)	−0.62 (−2.28)
Investigations	414	0.86 (0.78–0.95)	0.87 (8.51)	0.87 (0.8)	−0.2 (−1.87)
Cardiac disorders*	407	2.05 (1.86–2.27)	2 (208.64)	2 (1.84)	1 (−0.67)
Respiratory, thoracic and mediastinal disorders	401	1.1 (0.99–1.22)	1.09 (3.47)	1.09 (1.01)	0.13 (−1.54)
Musculoskeletal and connective tissue disorders	323	0.79 (0.71–0.88)	0.8 (17.57)	0.8 (0.73)	−0.33 (−1.99)
Skin and subcutaneous tissue disorders	192	0.45 (0.39–0.52)	0.47 (124.32)	0.47 (0.41)	−1.1 (−2.77)
Immune system disorders*	161	1.91 (1.63–2.23)	1.89 (68.34)	1.89 (1.66)	0.92 (−0.75)
Infections and infestations	155	0.37 (0.32–0.43)	0.38 (162.08)	0.38 (0.34)	−1.38 (−3.05)
Vascular disorders	147	0.89 (0.75–1.04)	0.89 (2.15)	0.89 (0.77)	−0.17 (−1.84)
Eye disorders	124	0.8 (0.67–0.96)	0.81 (5.9)	0.81 (0.69)	−0.31 (−1.98)
Renal and urinary disorders	108	0.76 (0.63–0.92)	0.76 (8.02)	0.76 (0.65)	−0.39 (−2.05)
Metabolism and nutrition disorders	67	0.4 (0.32–0.51)	0.41 (59.34)	0.41 (0.33)	−1.3 (−2.96)
Social circumstances*	47	1.42 (1.06–1.89)	1.41 (5.7)	1.41 (1.11)	0.5 (−1.17)
Product issues	46	0.38 (0.28–0.51)	0.38 (46.59)	0.38 (0.3)	−1.39 (−3.05)
Hepatobiliary disorders	39	0.55 (0.4–0.76)	0.56 (13.93)	0.56 (0.43)	−0.85 (−2.51)
Surgical and medical procedures	36	0.34 (0.25–0.48)	0.35 (44.78)	0.35 (0.26)	−1.53 (−3.19)
Reproductive system and breast disorders	34	0.54 (0.38–0.75)	0.54 (13.54)	0.54 (0.41)	−0.89 (−2.56)
Blood and lymphatic system disorders	34	0.26 (0.18–0.36)	0.26 (72.8)	0.26 (0.2)	−1.94 (−3.61)
Endocrine disorders*	29	1.49 (1.04–2.15)	1.49 (4.66)	1.49 (1.1)	0.57 (−1.09)
Ear and labyrinth disorders	28	0.85 (0.58–1.23)	0.85 (0.79)	0.85 (0.62)	−0.24 (−1.91)
Congenital, familial and genetic disorders	28	1.2 (0.83–1.74)	1.2 (0.91)	1.2 (0.88)	0.26 (−1.41)
Pregnancy, puerperium and perinatal conditions	19	0.57 (0.37–0.9)	0.58 (5.98)	0.58 (0.39)	−0.8 (−2.46)
Neoplasms benign, malignant and unspecified (incl cysts and polyps)	10	0.05 (0.03–0.09)	0.05 (189.19)	0.05 (0.03)	−4.35 (−6.01)

Asterisks (*) indicate statistically significant signals. ROR, reporting odds ratio; PRR, proportional reporting ratio; EBGM, empirical Bayesian geometric mean; EBGM05, the lower limit of the 95% confidence interval of EBGM; IC, information component; IC025, the lower limit of the 95% confidence interval of the IC.

### 3.4 Preferred term level distribution of AEs

**Figure 5:**
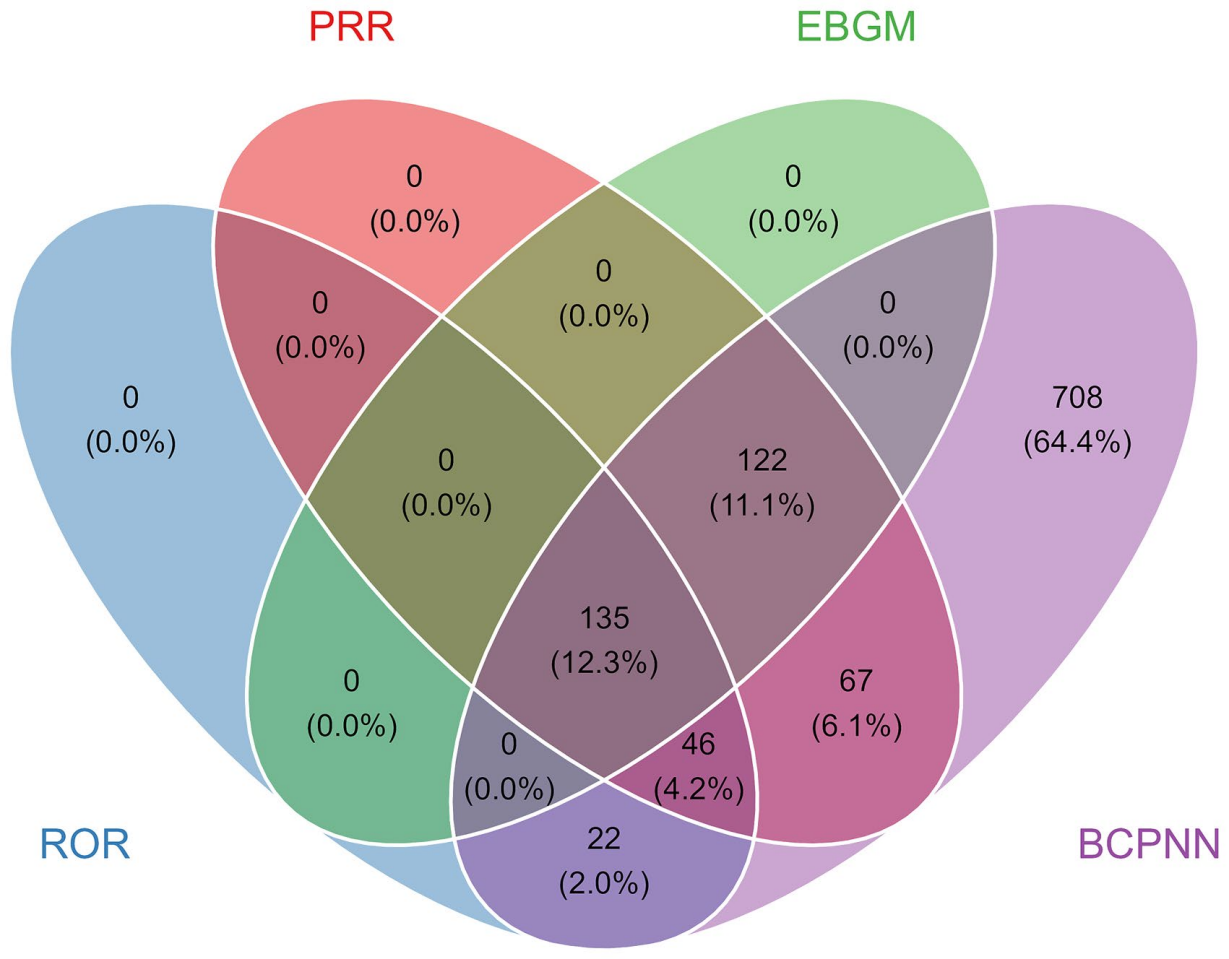
Venn diagram for the screening of all PTs based on the results of the four algorithms.

In our assessment, four different computational methods were employed at the PT level. These methods were utilized to evaluate adverse drug reactions and to determine their compliance with pre-established screening criteria. As a result, 1,100 PTs were identified. Figure 5 shows that 135 PTs satisfied the criteria set by all four computational methods. Table 4 presents the top 30 most frequently reported PTs, which are arranged according to the frequency of reports. Among this subset of the most commonly reported AEs, we detected positive signal reactions such as toxicity to various agents, completed suicide, drug abuse, cardiac arrest, respiratory arrest, drug hypersensitivity, dizziness, somnolence, overdose, drug interaction, confusional state, fall, intentional overdose, hypotension, cardiorespiratory arrest, delirium, hallucination, feeling abnormal, tachycardia, depressed level of consciousness, constipation, and coma.

**Table 4:** The top 30 signal strength of adverse events of cyclobenzaprine ranked by the number of case reports at the PTs level in FAERS database.

Preferred terms (PTs)	Case reports	ROR (95% CI)	PRR (95% CI)	EBGM (EBGM05)	IC (IC025)
Toxicity to various agents*	351	17.84 (16.03–19.86)	17.07 (5310.55)	17.03 (15.57)	4.09 (2.42)
Completed suicide*	337	32.42 (29.06–36.17)	31.04 (9767.51)	30.91 (28.2)	4.95 (3.28)
Drug abuse*	179	17.16 (14.79–19.9)	16.78 (2653.26)	16.74 (14.79)	4.07 (2.4)
Drug ineffective	171	1.03 (0.88–1.19)	1.03 (0.11)	1.03 (0.9)	0.04 (−1.63)
Cardiac arrest*	132	12.57 (10.58–14.94)	12.37 (1379.4)	12.35 (10.69)	3.63 (1.96)
Respiratory arrest*	126	34.7 (29.08–41.39)	34.14 (4035.79)	33.98 (29.32)	5.09 (3.42)
Drug hypersensitivity*	108	4.33 (3.58–5.23)	4.28 (272.18)	4.28 (3.65)	2.1 (0.43)
Dizziness*	103	1.64 (1.35–1.99)	1.63 (25.12)	1.63 (1.38)	0.7 (−0.96)
Somnolence*	98	3.87 (3.17–4.72)	3.83 (205.69)	3.83 (3.24)	1.94 (0.27)
Overdose*	96	3.39 (2.78–4.15)	3.36 (160.05)	3.36 (2.84)	1.75 (0.08)
Drug interaction*	82	4.06 (3.27–5.05)	4.03 (187.25)	4.03 (3.36)	2.01 (0.34)
Confusional state*	81	3.94 (3.16–4.9)	3.9 (175.35)	3.9 (3.25)	1.96 (0.3)
Nausea	65	0.65 (0.51–0.83)	0.65 (12.12)	0.65 (0.53)	−0.61 (−2.28)
Fall*	59	1.4 (1.08–1.81)	1.4 (6.65)	1.4 (1.13)	0.48 (−1.19)
Intentional overdose*	57	7.39 (5.69–9.59)	7.34 (312.31)	7.34 (5.9)	2.88 (1.21)
Fatigue	55	0.56 (0.43–0.73)	0.56 (19.07)	0.56 (0.45)	−0.83 (−2.5)
Hypotension*	55	2.16 (1.66–2.82)	2.15 (34.1)	2.15 (1.72)	1.11 (−0.56)
Cardio-respiratory arrest*	54	9.93 (7.6–12.98)	9.87 (430.12)	9.86 (7.88)	3.3 (1.63)
Headache	53	0.66 (0.5–0.87)	0.66 (9.11)	0.66 (0.53)	−0.59 (−2.26)
Delirium*	53	12.47 (9.52–16.34)	12.39 (554.28)	12.37 (9.87)	3.63 (1.96)
Hallucination*	52	5.6 (4.26–7.36)	5.57 (194.97)	5.56 (4.43)	2.48 (0.81)
Pain	51	0.64 (0.48–0.84)	0.64 (10.36)	0.64 (0.51)	−0.64 (−2.31)
Vomiting	50	0.85 (0.64–1.12)	0.85 (1.3)	0.85 (0.68)	−0.23 (−1.9)
Dyspnoea	50	0.69 (0.53–0.92)	0.7 (6.7)	0.7 (0.55)	−0.52 (−2.19)
Feeling abnormal*	49	1.56 (1.18–2.07)	1.56 (9.77)	1.56 (1.23)	0.64 (−1.03)
Tachycardia*	47	4.19 (3.15–5.59)	4.17 (113.52)	4.17 (3.28)	2.06 (0.39)
Depressed level of consciousness*	45	8.9 (6.64–11.93)	8.85 (313.29)	8.84 (6.92)	3.14 (1.48)
Constipation*	45	1.71 (1.27–2.29)	1.7 (13.04)	1.7 (1.33)	0.77 (−0.9)
Coma*	44	7.25 (5.39–9.75)	7.21 (235.45)	7.21 (5.62)	2.85 (1.18)
Malaise	43	0.76 (0.56–1.02)	0.76 (3.39)	0.76 (0.59)	−0.4 (−2.07)

Asterisks (*) indicate statistically significant signals. ROR, reporting odds ratio; PRR, proportional reporting ratio; EBGM, empirical Bayesian geometric mean; EBGM05, the lower limit of the 95% confidence interval of EBGM; IC, information component; IC025, the lower limit of the 95% confidence interval of the IC.

Our research findings are largely consistent with the adverse reactions mentioned in the prescribing information of cyclobenzaprine. Specifically, we identified several AEs that align with the known risk: cardiac arrest (*n* = 132, ROR 12.57 [10.58–14.94], PRR 12.37 [1379.4], EBGM 12.35 [10.69], IC 3.63 [1.96]), respiratory arrest (*n* = 126, ROR 34.7 [29.08–41.39], PRR 34.14 [4035.79], EBGM 33.98 [29.32], IC 5.09 [3.42]), drug hypersensitivity (*n* = 108, ROR 4.33 [3.58–5.23], PRR 4.28 [272.18], EBGM 4.28 [3.65], IC 2.1 [0.43]), dizziness (*n* = 103, ROR 1.64 [1.35–1.99], PRR 1.63 [25.12], EBGM 1.63 [1.38], IC 0.7 [−0.96]) and somnolence (*n* = 98, ROR 3.87 [3.17–4.72], PRR 3.83 [205.69], EBGM 3.83 [3.24], IC 1.94 [0.27]).

Apart from the adverse reactions stated in the drug’s prescribing information, a remarkable frequency of reports for other problems was observed, such as toxicity to various agents (*n* = 351, ROR 17.84 [16.03–19.86], PRR 17.07 [5310.55], EBGM 17.03 [15.57], IC 4.09 [2.42]), completed suicide (*n* = 337, ROR 32.42 [29.06–36.17], PRR 31.04 [9767.51], EBGM 30.91 [28.2], IC 4.95 [3.28]), drug abuse (*n* = 179, ROR 17.16 [14.79–19.9], PRR 16.78 [2653.26], EBGM 16.74 [14.79], IC 4.07 [2.4]), overdose (*n* = 96, ROR 3.39 [2.78–4.15], PRR 3.36 [160.05], EBGM 3.36 [2.84], IC 1.75 [0.08]), drug interaction (*n* = 82, ROR 4.06 [3.27–5.05], PRR 4.03 [187.25], EBGM 4.03 [3.36], IC 2.01 [0.34]) and confusional state (*n* = 81, ROR 3.94 [3.16–4.9], PRR 3.9 [175.35], EBGM 3.9 [3.25], IC 1.96 [0.3]). Among all PTs, toxicity to various agents and completed suicide were the most frequently reported AEs and were especially conspicuous.

**Figure 6:**
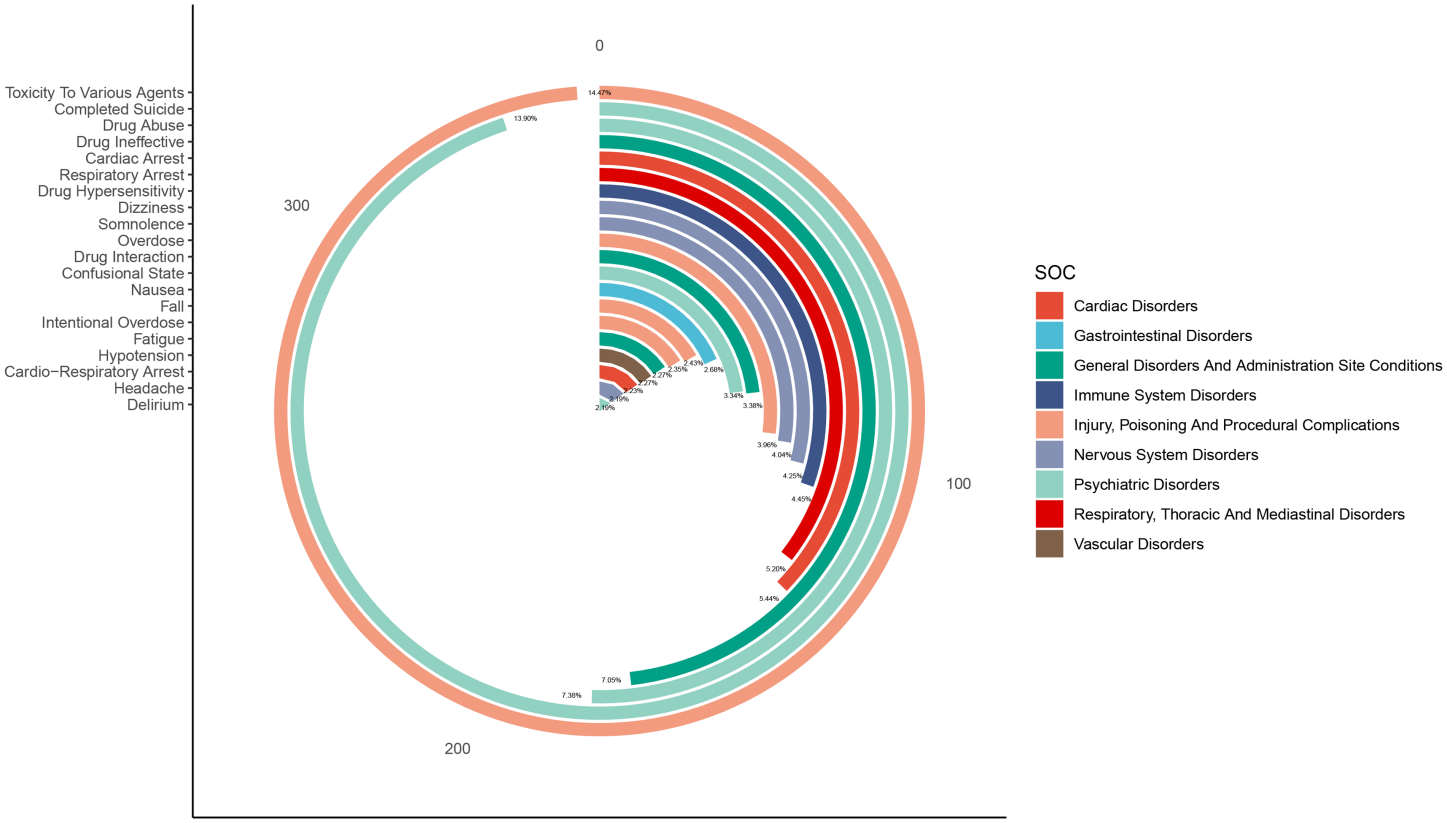
Signal strength of top 20 AEs of cyclobenzaprine at the PT Level in FAERS database.

**Figure 7:**
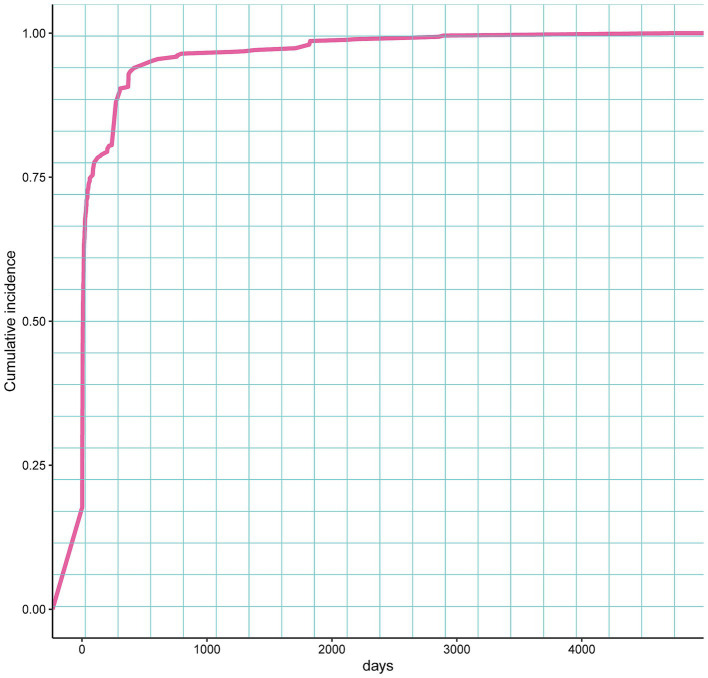
The progression of cumulative incidence of the reported PTs.

To more precisely clarify the relationship between these AEs and their SOC categorizations, Figure 6 shows the top 20 PTs along with their associated SOCs. During the observation period of the study, Figure 7 monitors the cumulative incidence of the reported PTs. This effectively highlights the pattern and frequency with which these AEs were recorded.

### 3.5 Subgroup analysis

In our study, significant demographic heterogeneity was observed, particularly in gender distribution. To mitigate the potential confounding effects of demographic characteristics on the results, we conducted a subgroup analysis stratified by gender. Within both the male and female subgroups, “completed suicide” and “toxicity to various agents” emerged as the two most frequently reported AEs. Among the 30 most common AEs meeting the positive signal criteria, certain events were exclusively observed in males, including delirium, hallucination, hypotension, urinary retention, tachycardia, constipation, mental status changes, heart rate increased, agitation, myoclonus, and serotonin syndrome (as shown in Supplementary Table 3). In contrast, drug hypersensitivity, loss of consciousness, dry mouth, tremor, and muscle spasms were specific to females (Supplementary Table 4). Notably, although “fall” ranked among the top 30 reported AEs in terms of case numbers, it only met the positive signal criteria in the male subgroup.

### 3.6 Sensitivity analysis

During the clinical application of cyclobenzaprine, it is often noticed that this drug is frequently prescribed concurrently with other common pain-relieving medications, like ibuprofen, naproxen, and acetaminophen, as well as a diverse range of other drugs. After excluding reports involving the concurrent use of other drugs, our research detected 992 individual reports, which accounted for 1,951 distinct AEs. The potential persistent adverse reactions encompassed completed suicide, toxicity to various agents, drug abuse, drug ineffectiveness, drug hypersensitivity, cardiac arrest, drowsiness, respiratory arrest, dizziness, overdose, confusional state, drug interaction, fall, intentional overdose, hallucination, abnormal sensation, delirium, cardio-respiratory arrest, tachycardia, serotonin syndrome, dry mouth, increased heart rate, and hypotension (Supplementary Table 5).

## 4 Discussion

Despite numerous reports on cyclobenzaprine, researches of its side effects based on large samples are limited. Our study, using a large dataset, comprehensively assessed AEs associated with cyclobenzaprine since 2004 (cyclobenzaprine was first introduced to the market in 1977. However, the FAERS database has records cyclobenzaprine starting from 2004. Consequently, our study commenced in 2004.). This study, by analyzing FAERS data, confirmed previously recognized adverse reactions listed on the cyclobenzaprine drug label, including psychiatric and nervous system disorders (delirium, hallucination, dizziness, somnolence, feeling abnormal and depressed level of consciousness) cardiac disorders (hypotension and tachycardia) and constipation, etc. Also, we find some AEs not cited on the label, such as toxicity to various agents, completed suicide, drug abuse, overdose, drug interaction, and confusional state. Thus, in clinical practice, it is essential to closely monitor adverse reactions when using cyclobenzaprine for treatment.

There have been many prior clinical trials of adverse reactions to the label. In a five-year multicenter retrospective review of cyclobenzaprine toxicity by H A Spiller et al., the common effects were lethargy in 216 cases (54%) and sinus tachycardia in 132 cases (33%). Among adults (age > 10 years), sinus tachycardia was generally mild, with only 11 patients having a Maximum Recorded Heart Rate (MHR) exceeding 140 beats per minute (bpm). In contrast, for a child less than 10 years old, the highest MHR recorded was 180 bpm (in a 3-year-old). Moreover, the average MHR for all tachycardiac children (*n* = 4) was 157 bpm (±22) (35). Notably, cyclobenzaprine’s cardiac effects extend beyond tachycardia, with distinct impacts on cardiac muscle physiology and pathology that are relevant to its use in MSK pain (36, 37). Physiologically, in isolated rabbit atria, non-cardiotoxic concentrations (≤9 × 10^−7^ M) of cyclobenzaprine block muscarinic cholinergic receptors, augment norepinephrine-mediated adrenergic responses, and exhibit antihistaminic activity by slowing histamine-induced rate increases with poorly reversible inhibition (38). Pathologically, it shares tricyclic antidepressant-like sodium channel-blocking properties: even at therapeutic concentrations (16 ng/mL), it may contribute to QRS prolongation and conduction abnormalities, while high concentrations (>3.6 × 10^−6^ M) cause irreversible cardiac depression (39, 40). During MSK-associated pain, this becomes clinically relevant—MSK pain triggers sympathetic activation (elevating heart rate and myocardial oxygen demand), which synergizes with cyclobenzaprine’s adrenergic potentiation and tachycardia risk, amplifying cardiac stress (41).

Cyclobenzaprine exhibits potential effects on cardiac inflammation and acute respiratory inflammatory stages (e.g., ARDS), though these are primarily derived from preclinical studies and clinical case observations rather than direct therapeutic evidence (42). In terms of cardiac inflammation, cyclobenzaprine, as a tricyclic derivative, shares structural similarities with tricyclic antidepressants and can modulate inflammatory signaling pathways: it downregulates the TLR4/MyD88/NF-κB pathway—key in mediating cardiac inflammatory responses—to reduce the expression of pro-inflammatory cytokines and inhibit inflammatory cell infiltration, which may indirectly alleviate cardiac inflammatory damage secondary to MSK pain-related stress (42–44). In acute respiratory inflammatory stages like ARDS, cyclobenzaprine’s effects are twofold: on one hand, it relaxes airway smooth muscle by inhibiting L-type voltage-dependent Ca^2+^ channels (L-VDCC) and nonselective cation channels (NSCC), reduces pulmonary inflammatory cell accumulation, and downregulates the PI3K/AKT/mTOR pathway to alleviate airway inflammation—effects that may indirectly mitigate respiratory inflammatory exacerbation; on the other hand, clinical cases of polypharmacy overdose have linked it to ARDS development, possibly due to synergistic cardiac depression and impaired pulmonary perfusion, highlighting its risk in complex inflammatory states (36, 44). Thus, while cyclobenzaprine lacks direct anti-inflammatory indications for cardiac or ARDS, its modulatory effects on inflammatory pathways and ion channels warrant cautious use in patients with comorbid cardiac or respiratory inflammatory conditions.

Additionally, Toth PP et al.’s review noted that AEs associated with muscle relaxants like cyclobenzaprine often involve the central nervous system, including somnolence and dizziness, of these, somnolence was the most frequent problem encountered by patients of all the adverse effects (45). In addition, in the treatment of acute skeletal muscle spasms with low-dose cyclobenzaprine hydrochloride regimen conducted by David G Borenstein et al., Somnolence was the most common adverse reaction, and the incidence of Somnolence increased by 19.7% with high-dose cyclobenzaprine (18). Moreover, in a pharmacokinetic comparison of cyclobenzaprine conducted by A Randomized et al., somnolence was the most frequent AE (46). The results of this study share some similarities with previous research. Although somnolence was not the most common adverse reaction in this study, its frequency of occurrence ranked ninth among all adverse reactions, with a relatively high proportion. The reasons for the occurrence of drowsiness after taking cyclobenzaprine is likely mediated by functional antagonism of central H1R, Histamine, the endogenous agonist of H1R, elicited a sigmoidal dose–response curve of Ca^2+^ luminescence in HEK293 cells transfected with H1R, with a pEC50 of 6.1 ± 0.1 (764 NM). Cyclobenzaprine right-shifted the histamine dose–response curve, with pEC50 shifts to 4.1 (100 PM), 2.3 (1 NM), and 0.14 (10 NM). Also, cyclobenzaprine significantly reduced the Emax of the histamine-induced effect to 60% (100 PM), 30% (1 NM), and 15% (10 NM), indicating noncompetitive inhibition. Finally, the calculated pA2 values for cyclobenzaprine and diphenhydramine were 11.92 (1.2 PM) and 10.15 (70 PM), respectively, showing that cyclobenzaprine is approximately 70-fold more potent than diphenhydramine, suggesting an even more significant antihistaminergic response. There will be more adverse reactions to somnolence (47). Therefore, when using cyclobenzaprine for daily treatment, pay attention to observing this adverse reaction.

In the SOC level AEs report in this study, psychiatric disorders had the highest proportion of 1,260 cases, corresponding to the symptoms in PT: confusional state, delirium, and hallucination. Because cyclobenzaprine exhibits a cyclic structure that bears resemblance to amitriptyline. Amitriptyline, a tricyclic antidepressant, also functions as a skeletal muscle relaxant (48, 49). The two drugs share an identical cyclic structure. Drugs of this type exert their effects by virtue of their potent binding ability and antagonistic activity at 5-HT2A, α1 – adrenergic, H1 – histaminergic, and M1 – muscarinic receptors (50). Among them, the 5-HT2A receptor is widely distributed in multiple regions of the brain, including the frontal lobe, temporal lobe, cingulate gyrus, etc. (51). These brain regions are closely associated with advanced neural activities such as cognitive function, emotional regulation, and consciousness state (52). Cyclobenzaprine has a high affinity for the 5-HT2A receptor (53). Once the drug molecules bind to the 5-HT2A receptor, the normal signal transmission of 5 – hydroxytryptamine is blocked (54). Under normal physiological conditions, serotonin participates in regulating the release of neurotransmitters, the excitability of neurons, and neural plasticity by binding to the 5-HT2A receptor (55). However, the action of cyclobenzaprine disrupts this balance, leading to the dysfunction of relevant neural circuits. As a result, adverse reactions such as Confusional State, Delirium, and Hallucination, which are related to psychiatric symptoms, occur. In clinical practice, we should first conduct a comprehensive pre-treatment mental assessment of patients. Close monitoring of patients’ mental status during treatment is essential, including regular evaluations of cognitive function, mood, and consciousness. If early signs of psychiatric symptoms emerge, appropriate interventions should be taken promptly, such as adjusting the dosage of cyclobenzaprine, considering alternative medications, or consulting a psychiatrist for collaborative management.

Another interesting phenomenon is the abuse and overdose and completed suicide of drugs with high levels in our PT. As mentioned earlier, cyclobenzaprine and amitriptyline share a similar structure, with the difference of one double bond. And amitriptyline is not infrequently ingested in excessive amounts in cases of self-poisoning attempts. And, amitriptyline, this medication, which is used to treat a patient’s major depressive disorder (MDD), during the initial stage of drug use, the drug may act on specific neural pathways, triggering a series of physiological reactions such as activation effects. These reactions can, in turn, induce symptoms like anxiety and akathisia (56). These symptoms are likely to disrupt the patient’s emotional state, causing distress and impulsivity, thereby increasing the risk of suicidal behavior. From the perspective of the drug’s mechanism of action, the drug exhibits a certain affinity for the muscarinic, histaminergic, and adrenergic systems. This affinity gives rise to a series of associated side-effects, including constipation, dry mouth, dry eyes, and restlessness (57). These side-effects not only cause physical discomfort to the patient but may also interfere with the normal emotional regulation mechanisms of the patient, leading to a deterioration of the emotional experience. Moreover, the worsening of the emotional state further elevates the patient’s risk of suicide (58). Meanwhile, as a commonly used medication for MSK pain such as lower back pain and neck pain, cyclobenzaprine accounts for nearly one-third of muscle relaxant prescriptions (59, 60). Research has shown that when 20 to 80 milligrams of cyclobenzaprine is used for recreational purposes, this dosage can cause significant drowsiness and relaxation, brings a sense of euphoria and pleasant experience to drug users (61). Cyclobenzaprine’s potential to induce drug-related adverse effects, such as overdose and addictive behaviors, is closely tied to its impact on the brain’s reward system. Research has established that dopamine is pivotal in the brain’s reward circuitry. When cyclobenzaprine acts on this system, it elicits reward—like responses through dopamine and other neurotransmitters. However, long-term use of cyclobenzaprine can lead to neuroadaptations in dopaminergic neurons and associated neural pathways (62). This process, which involves multiple neurotransmitter systems, causes alterations in synapse structure and function, thereby giving rise to abnormal neural circuits that are strongly linked to addictive behaviors. For instance, the strengthening of conditioned reflexes means that cues related to pharmaceuticals can easily trigger dopamine release, fueling the motivation to seek the drug. Additionally, individuals who have been using cyclobenzaprine for a long time often exhibit reduced sensitivity to both drug-related and non-drug rewards (63). Simultaneously, chronic use of pharmaceuticals impairs the function of the prefrontal cortex, which is responsible for self-regulation, thus weakening an individual’s ability to control their drug-seeking behavior (62). The cumulative effect of these factors disrupts the normal functioning of the brain’s reward system. This provides an explanation for the relatively high incidence of cyclobenzaprine overdose and abuse cases observed in the PT analysis. Moreover, cyclobenzaprine exhibits anticholinergic and antihistaminergic effects. Richelson previously suggested that the anticholinergic and antihistamine effects of tricyclic drugs might underlie their abuse potential (64). Thus, cyclobenzaprine’s antihistaminergic and anticholinergic properties may synergistically contribute to its abuse liability. However, several case studies have shown that these properties can induce euphoric or psychedelic effects, which may drive user abuse (65–68). Consequently, when prescribing this drug to those with psychiatric disorders and individuals at risk of drug abuse, comprehensive risk–benefit assessments should be meticulously carried out, and close monitoring during the treatment course is essential to minimize potential adverse outcomes and ensure the safe and rational use of cyclobenzaprine (61).

The results of the subgroup analysis underscored that, in addition to typical somatic ailments, mental manifestations in male patients, such as delirium, hallucination, changes in mental status, and serotonin syndrome, warrant particular consideration. In contrast, female patients should be closely monitored for the potential risks associated with drug hypersensitivity, loss of consciousness, dry mouth, tremor, and muscle spasms. Significantly, when male patients are administered this drug, it is strongly advisable to initiate interdisciplinary cooperation with psychiatrists (69, 70). This collaborative strategy enables the early detection and intervention of patients’ mental symptoms, thus averting the aggravation of the disease condition (71). In the sensitivity analysis, we identified potential adverse reactions that are persistently associated with cyclobenzaprine monotherapy. These encompass urinary retention, changes in mental status, elevated heart rate, agitation, myoclonus, and serotonin syndrome. Among them, while urinary retention, alterations in mental status, increased heart rate, agitation, and myoclonus are non-lethal yet impactful AEs that can undermine treatment adherence and thereby negatively affect the therapeutic efficacy, serotonin syndrome is a severe and potentially life-threatening adverse reaction. This meticulous monitoring can contribute to optimizing treatment outcomes and enhancing the effectiveness of the medication, especially when dealing with the highly dangerous serotonin syndrome, early detection and intervention are crucial to prevent fatal consequences.

Beyond the primary analysis, our research integrated a time-based assessment of AEs. We made use of the Weibull distribution model to predict the incidence of these events. This method is beneficial for establishing effective monitoring time intervals for adverse reactions related to drug treatment. The results emphasize the importance of strict monitoring, particularly during the first month after starting cyclobenzaprine therapy. Concentrating on this early-detection period is crucial for quickly recognizing and dealing with potential AEs, with the ultimate goal of improving patient safety and the success of treatment.

Although this study provides a comprehensive analysis of the safety profile of cyclobenzaprine, several limitations should be acknowledged. First, the statistical methods utilized, such as ROR, PRR, EBGM, and BCPNN, indicate potential associations but do not confirm direct causal relationships between the drug and AEs (27). Therefore, additional prospective research is necessary to establish causality. Second, the FAERS database, which depends on voluntary submissions, is susceptible to reporting biases, including under-reporting and over-reporting. Data submitted by consumers may lack the accuracy and thoroughness typically provided by healthcare professionals (72). Third, the absence of detailed clinical information, including medication adherence and dosing specifics, further limits the interpretation of the findings. Additionally, unmeasured confounding variables—including age-specific physiological differences, racial disparities in drug metabolism, comorbidities such as cardiovascular or psychiatric disorders, and potential drug–drug interactions with concurrent medications (e.g., opioids or antidepressants)—may have influenced the observed AE patterns, as these factors are known to modulate drug safety profiles in real-world settings. Despite these constraints, the results offer valuable insights for clinicians to enhance patient monitoring and identify potential AEs associated with cyclobenzaprine therapy.

## 5 Conclusion

On the basis of a comprehensive consideration, a meticulous evaluation of the safety profile of cyclobenzaprine was executed within the clinical setting. Through meticulous examination of the data derived from the FAERS database, the frequency of AEs and their precise onset timings were determined. Our analysis not only corroborated the previously acknowledged AEs but also revealed additional potential reactions that were not explicitly delineated on the product label, such as toxicity to various agents, completed suicide, drug abuse, overdose, drug interaction, and confusional state. These novel insights are clinically significant as they prompt more rigorous safety surveillance during cyclobenzaprine treatment, enabling early detection and mitigation of AEs, while also guiding evidence based clinical decision making and future research.

## Data Availability

The datasets presented in this study can be found in online repositories. The names of the repository/repositories and accession number(s) can be found in the article/Supplementary material.
