# Supplementary material for: Cyclobenzaprine-related adverse events: a comprehensive pharmacovigilance analysis using the FDA Adverse Event Reporting System
Source: Front Med (Lausanne). 2025 Sep 22;12:1574395. doi: 10.3389/fmed.2025.1574395 (PMC12497723; doi:10.3389/fmed.2025.1574395)
Supplement: Supplementary file 1 [file Data_Sheet_1.docx]

**Supplementary Table 1:** Ratio imbalance measurement algorithm.

| **Characteristics** | **AEs of interest** | **Other AEs** | **Total** |
| --- | --- | --- | --- |
| **Target drug** | a | b | a + b |
| **Other drugs** | c | d | c + d |
| **Total** | a + c | b + d | N = a + b + c + d |

a: Number of reports that contain both targeted drug and targeted drug adverse reactions; b: Number of reports of other drug adverse reactions that contain the targeted drug; c: Number of reports of targeted drug adverse reactions that contain other drugs; d: Number of reports that contain other drugs and other drug adverse reactions; All other drugs, all drugs in the FAERS, dataset other than teprotumumab.

**Supplementary Table 2:** Summary of major algorithms used for signal detection.

| **Method** | **Formula** | **Threshold** |
| --- | --- | --- |
| **ROR** | ROR = ad/b/c  95%CI = e ^ln (ROR)±1.96(l/a+1/b+l/c+l/d) ^0.5^ | a ≥ 3  ROR ≥ 3  95%CI (lower limit) > 1 |
| **PRR** | PRR = a (c + d)/c/(a+b)  x^2^ = [(ad-bc) ^2] (a+b + c + d)/[(a+b) (c + d) (a+c) (b + d) | a ≥ 3  PRR ≥ 2  95%CI (lower limit) > 1 |
| **BCPNN** | IC = logza (a+b + c + d)/((a+c) (a+b))  95%Cl = E(IC)±2V(IC) ^0.5 | IC025 > 0 |
| **MGPS** | EBGM = a (a+b + c + d)/(a+c)/(a+b)  95%CI = e ^ln (EBGM)±1.96(l/a+l/b+l/c+l/d) ^0.5^ | EBGM05 > 2 |

ROR: reporting odds ratio; PRR, proportional reporting ratio; BCPNN: bayesian confidence propagation neural network; MGPS: Multi-item gamma Poisson shrinker; 95% CI: 95% confidence interval; EBGM: empirical Bayesian geometric mean.

**Supplementary Table 3:** Top 30 most frequent adverse events for Cyclobenzaprine in males at the PT level from FAERS data.

| **Preferred terms (PTs)** | **Case reports** | **ROR (95% CI)** | | **PRR (95% CI)** | **EBGM**  **(EBGM05)** | **IC(IC025)** |
| --- | --- | --- | --- | --- | --- | --- |
| **Completed Suicide*** | 100 | | 32.44 ( 26.54 - 39.65 ) | 31.05 ( 2908.4 ) | 31.01 ( 26.22 ) | 4.95 ( 3.29 ) |
| **Toxicity To Various Agents*** | 100 | | 17.13 ( 14.02 - 20.93 ) | 16.41 ( 1450.42 ) | 16.4 ( 13.87 ) | 4.04 ( 2.37 ) |
| **Drug Abuse*** | 79 | | 25.88 ( 20.67 - 32.39 ) | 25.01 ( 1821.28 ) | 24.98 ( 20.7 ) | 4.64 ( 2.98 ) |
| **Cardiac Arrest*** | 44 | | 14.19 ( 10.53 - 19.12 ) | 13.93 ( 528.52 ) | 13.92 ( 10.85 ) | 3.8 ( 2.13 ) |
| **Confusional State*** | 41 | | 6.79 ( 4.98 - 9.24 ) | 6.68 ( 198.52 ) | 6.68 ( 5.16 ) | 2.74 ( 1.07 ) |
| **Respiratory Arrest*** | 39 | | 36.22 ( 26.39 - 49.73 ) | 35.62 ( 1310.76 ) | 35.56 ( 27.28 ) | 5.15 ( 3.49 ) |
| **Drug Ineffective** | 37 | | 0.75 ( 0.54 - 1.03 ) | 0.75 ( 3.14 ) | 0.75 ( 0.57 ) | -0.41 ( -2.08 ) |
| **Dizziness*** | 37 | | 1.99 ( 1.44 - 2.76 ) | 1.98 ( 18 ) | 1.98 ( 1.51 ) | 0.98 ( -0.68 ) |
| **Overdose*** | 29 | | 3.47 ( 2.4 - 5 ) | 3.44 ( 50.26 ) | 3.44 ( 2.53 ) | 1.78 ( 0.11 ) |
| **Delirium*** | 29 | | 23.19 ( 16.08 - 33.46 ) | 22.91 ( 607.33 ) | 22.89 ( 16.84 ) | 4.52 ( 2.85 ) |
| **Somnolence*** | 25 | | 3.33 ( 2.25 - 4.94 ) | 3.3 ( 40.3 ) | 3.3 ( 2.38 ) | 1.72 ( 0.06 ) |
| **Hallucination*** | 25 | | 9.14 ( 6.16 - 13.56 ) | 9.05 ( 179.13 ) | 9.05 ( 6.5 ) | 3.18 ( 1.51 ) |
| **Hypotension*** | 23 | | 3.07 ( 2.03 - 4.62 ) | 3.05 ( 31.69 ) | 3.04 ( 2.16 ) | 1.61 ( -0.06 ) |
| **Urinary Retention*** | 21 | | 17.08 ( 11.11 - 26.25 ) | 16.93 ( 314.7 ) | 16.92 ( 11.81 ) | 4.08 ( 2.41 ) |
| **Tachycardia*** | 20 | | 6.05 ( 3.89 - 9.39 ) | 6 ( 83.51 ) | 6 ( 4.15 ) | 2.59 ( 0.92 ) |
| **Drug Interaction*** | 20 | | 3.34 ( 2.15 - 5.19 ) | 3.32 ( 32.56 ) | 3.32 ( 2.3 ) | 1.73 ( 0.07 ) |
| **Constipation*** | 20 | | 2.57 ( 1.65 - 3.99 ) | 2.56 ( 19.01 ) | 2.56 ( 1.77 ) | 1.35 ( -0.31 ) |
| **Fall*** | 20 | | 1.6 ( 1.03 - 2.49 ) | 1.6 ( 4.52 ) | 1.6 ( 1.11 ) | 0.68 ( -0.99 ) |
| **Intentional Overdose*** | 18 | | 7.89 ( 4.96 - 12.55 ) | 7.84 ( 107.41 ) | 7.83 ( 5.31 ) | 2.97 ( 1.3 ) |
| **Mental Status Changes*** | 18 | | 17.22 ( 10.83 - 27.39 ) | 17.1 ( 272.7 ) | 17.08 ( 11.59 ) | 4.09 ( 2.43 ) |
| **Depressed Level Of Consciousness*** | 17 | | 11.38 ( 7.06 - 18.34 ) | 11.3 ( 159.65 ) | 11.3 ( 7.58 ) | 3.5 ( 1.83 ) |
| **Heart Rate Increased*** | 16 | | 4.33 ( 2.65 - 7.08 ) | 4.31 ( 40.66 ) | 4.3 ( 2.85 ) | 2.11 ( 0.44 ) |
| **Dyspnoea** | 16 | | 0.75 ( 0.46 - 1.23 ) | 0.75 ( 1.31 ) | 0.75 ( 0.5 ) | -0.41 ( -2.08 ) |
| **Feeling Abnormal*** | 16 | | 1.72 ( 1.05 - 2.82 ) | 1.72 ( 4.82 ) | 1.72 ( 1.14 ) | 0.78 ( -0.89 ) |
| **Agitation*** | 16 | | 5.68 ( 3.47 - 9.29 ) | 5.65 ( 61.27 ) | 5.65 ( 3.74 ) | 2.5 ( 0.83 ) |
| **Cardio-Respiratory Arrest*** | 16 | | 9.94 ( 6.08 - 16.26 ) | 9.88 ( 127.72 ) | 9.88 ( 6.54 ) | 3.3 ( 1.64 ) |
| **Myoclonus*** | 15 | | 33.62 ( 20.23 - 55.89 ) | 33.41 ( 470.97 ) | 33.36 ( 21.81 ) | 5.06 ( 3.39 ) |
| **Nausea** | 14 | | 0.47 ( 0.28 - 0.8 ) | 0.48 ( 8.19 ) | 0.48 ( 0.31 ) | -1.07 ( -2.74 ) |
| **Serotonin Syndrome*** | 14 | | 20.16 ( 11.92 - 34.1 ) | 20.04 ( 253.11 ) | 20.02 ( 12.9 ) | 4.32 ( 2.66 ) |
| **Fatigue** | 14 | | 0.48 ( 0.28 - 0.81 ) | 0.48 ( 7.83 ) | 0.48 ( 0.31 ) | -1.05 ( -2.72 ) |

Asterisks (*) indicate statistically significant signals. Abbreviations: ROR, reporting odds ratio; PRR, proportional reporting ratio; EBGM, empirical Bayesian geometric mean; EBGM05, the lower limit of the 95% confidence interval of EBGM; IC, information component; IC025, the lower limit of the 95% confidence interval of the IC.

**Supplementary Table 4:** Top 30 most frequent adverse events for Cyclobenzaprine in females at the PT level from FAERS data.

| **Preferred terms (PTs)** | **Case reports** | **ROR (95% CI)** | | **PRR (95% CI)** | **EBGM**  **(EBGM05)** | **IC(IC025)** |
| --- | --- | --- | --- | --- | --- | --- |
| **Completed Suicide*** | 191 | | 32.28 ( 27.91 - 37.32 ) | 30.9 ( 5520.49 ) | 30.83 ( 27.3 ) | 4.95 ( 3.28 ) |
| **Toxicity To Various Agents*** | 177 | | 15.73 ( 13.53 - 18.28 ) | 15.13 ( 2339.02 ) | 15.11 ( 13.32 ) | 3.92 ( 2.25 ) |
| **Drug Ineffective** | 100 | | 1.06 ( 0.87 - 1.29 ) | 1.06 ( 0.3 ) | 1.06 ( 0.89 ) | 0.08 ( -1.59 ) |
| **Drug Abuse*** | 90 | | 15.12 ( 12.27 - 18.64 ) | 14.83 ( 1160.94 ) | 14.81 ( 12.44 ) | 3.89 ( 2.22 ) |
| **Cardiac Arrest*** | 77 | | 12.9 ( 10.3 - 16.17 ) | 12.69 ( 829.78 ) | 12.68 ( 10.5 ) | 3.66 ( 2 ) |
| **Respiratory Arrest*** | 75 | | 36.31 ( 28.89 - 45.63 ) | 35.7 ( 2523.28 ) | 35.6 ( 29.4 ) | 5.15 ( 3.49 ) |
| **Somnolence*** | 64 | | 4.45 ( 3.48 - 5.7 ) | 4.4 ( 168.88 ) | 4.4 ( 3.58 ) | 2.14 ( 0.47 ) |
| **Dizziness*** | 59 | | 1.65 ( 1.28 - 2.13 ) | 1.64 ( 14.88 ) | 1.64 ( 1.32 ) | 0.71 ( -0.95 ) |
| **Overdose*** | 55 | | 3.42 ( 2.62 - 4.47 ) | 3.39 ( 93.09 ) | 3.39 ( 2.71 ) | 1.76 ( 0.1 ) |
| **Drug Hypersensitivity*** | 53 | | 3.73 ( 2.84 - 4.89 ) | 3.7 ( 104.51 ) | 3.69 ( 2.95 ) | 1.89 ( 0.22 ) |
| **Drug Interaction*** | 52 | | 4.54 ( 3.45 - 5.97 ) | 4.5 ( 141.76 ) | 4.5 ( 3.58 ) | 2.17 ( 0.5 ) |
| **Nausea** | 43 | | 0.76 ( 0.56 - 1.02 ) | 0.76 ( 3.28 ) | 0.76 ( 0.59 ) | -0.39 ( -2.06 ) |
| **Vomiting** | 39 | | 1.17 ( 0.85 - 1.61 ) | 1.17 ( 0.97 ) | 1.17 ( 0.9 ) | 0.23 ( -1.44 ) |
| **Headache** | 36 | | 0.79 ( 0.57 - 1.1 ) | 0.79 ( 1.95 ) | 0.79 ( 0.6 ) | -0.33 ( -2 ) |
| **Death** | 36 | | 0.58 ( 0.42 - 0.81 ) | 0.59 ( 10.51 ) | 0.59 ( 0.45 ) | -0.77 ( -2.43 ) |
| **Confusional State*** | 35 | | 2.98 ( 2.14 - 4.16 ) | 2.97 ( 45.79 ) | 2.97 ( 2.25 ) | 1.57 ( -0.1 ) |
| **Fatigue** | 35 | | 0.63 ( 0.45 - 0.87 ) | 0.63 ( 7.76 ) | 0.63 ( 0.48 ) | -0.67 ( -2.34 ) |
| **Cardio-Respiratory Arrest*** | 33 | | 10.68 ( 7.58 - 15.05 ) | 10.61 ( 287.17 ) | 10.6 ( 7.96 ) | 3.41 ( 1.74 ) |
| **Fall** | 32 | | 1.33 ( 0.94 - 1.89 ) | 1.33 ( 2.66 ) | 1.33 ( 1 ) | 0.41 ( -1.25 ) |
| **Pain** | 31 | | 0.68 ( 0.48 - 0.97 ) | 0.69 ( 4.51 ) | 0.69 ( 0.51 ) | -0.54 ( -2.21 ) |
| **Intentional Overdose*** | 30 | | 6.84 ( 4.78 - 9.79 ) | 6.8 ( 148.44 ) | 6.8 ( 5.03 ) | 2.76 ( 1.1 ) |
| **Malaise** | 28 | | 0.87 ( 0.6 - 1.26 ) | 0.87 ( 0.57 ) | 0.87 ( 0.64 ) | -0.21 ( -1.87 ) |
| **Feeling Abnormal*** | 27 | | 1.51 ( 1.04 - 2.21 ) | 1.51 ( 4.65 ) | 1.51 ( 1.1 ) | 0.59 ( -1.07 ) |
| **Loss Of Consciousness*** | 27 | | 2.91 ( 1.99 - 4.25 ) | 2.9 ( 33.59 ) | 2.9 ( 2.11 ) | 1.53 ( -0.13 ) |
| **Dry Mouth*** | 26 | | 4.55 ( 3.09 - 6.68 ) | 4.52 ( 71.45 ) | 4.52 ( 3.28 ) | 2.18 ( 0.51 ) |
| **Tremor*** | 26 | | 2.14 ( 1.46 - 3.15 ) | 2.13 ( 15.72 ) | 2.13 ( 1.55 ) | 1.09 ( -0.57 ) |
| **Asthenia** | 26 | | 0.96 ( 0.65 - 1.41 ) | 0.96 ( 0.05 ) | 0.96 ( 0.69 ) | -0.06 ( -1.73 ) |
| **Diarrhoea** | 26 | | 0.57 ( 0.39 - 0.83 ) | 0.57 ( 8.56 ) | 0.57 ( 0.41 ) | -0.81 ( -2.48 ) |
| **Dyspnoea** | 25 | | 0.61 ( 0.41 - 0.9 ) | 0.61 ( 6.19 ) | 0.61 ( 0.44 ) | -0.71 ( -2.37 ) |
| **Depressed Level Of Consciousness*** | 25 | | 8.7 ( 5.87 - 12.89 ) | 8.65 ( 169.17 ) | 8.65 ( 6.22 ) | 3.11 ( 1.45 ) |

Asterisks (*) indicate statistically significant signals. Abbreviations: ROR, reporting odds ratio; PRR, proportional reporting ratio; EBGM, empirical Bayesian geometric mean; EBGM05, the lower limit of the 95% confidence interval of EBGM; IC, information component; IC025, the lower limit of the 95% confidence interval of the IC.

**Supplementary Table 5:** Top 30 most frequent adverse events for Cyclobenzaprine excluding common medication co-usage at the PT level from FAERS data.

| **Preferred terms (PTs)** | **Case reports** | **ROR (95% CI)** | | **PRR (95% CI)** | **EBGM**  **(EBGM05)** | **IC(IC025)** |
| --- | --- | --- | --- | --- | --- | --- |
| **Completed Suicide*** | 236 | | 31.83 ( 27.93 - 36.27 ) | 30.49 ( 6720.25 ) | 30.4 ( 27.25 ) | 4.93 ( 3.26 ) |
| **Toxicity To Various Agents*** | 233 | | 16.57 ( 14.53 - 18.89 ) | 15.9 ( 3257.01 ) | 15.88 ( 14.22 ) | 3.99 ( 2.32 ) |
| **Drug Abuse*** | 149 | | 20.13 ( 17.1 - 23.7 ) | 19.61 ( 2629.76 ) | 19.57 ( 17.08 ) | 4.29 ( 2.62 ) |
| **Drug Ineffective*** | 148 | | 1.25 ( 1.06 - 1.48 ) | 1.25 ( 7.4 ) | 1.25 ( 1.09 ) | 0.32 ( -1.35 ) |
| **Drug Hypersensitivity*** | 105 | | 5.94 ( 4.9 - 7.21 ) | 5.84 ( 422.77 ) | 5.84 ( 4.97 ) | 2.55 ( 0.88 ) |
| **Cardiac Arrest*** | 99 | | 13.25 ( 10.86 - 16.17 ) | 13.03 ( 1099.52 ) | 13.01 ( 11.02 ) | 3.7 ( 2.04 ) |
| **Somnolence*** | 83 | | 4.61 ( 3.71 - 5.73 ) | 4.56 ( 231.22 ) | 4.56 ( 3.8 ) | 2.19 ( 0.52 ) |
| **Respiratory Arrest*** | 83 | | 32.01 ( 25.76 - 39.77 ) | 31.54 ( 2447.57 ) | 31.44 ( 26.22 ) | 4.97 ( 3.31 ) |
| **Dizziness*** | 74 | | 1.65 ( 1.31 - 2.08 ) | 1.64 ( 18.74 ) | 1.64 ( 1.36 ) | 0.72 ( -0.95 ) |
| **Overdose*** | 63 | | 3.13 ( 2.44 - 4.01 ) | 3.1 ( 89.98 ) | 3.1 ( 2.52 ) | 1.63 ( -0.03 ) |
| **Confusional State*** | 59 | | 4.03 ( 3.12 - 5.2 ) | 3.99 ( 132.71 ) | 3.99 ( 3.22 ) | 2 ( 0.33 ) |
| **Drug Interaction*** | 56 | | 3.9 ( 2.99 - 5.07 ) | 3.87 ( 119.23 ) | 3.86 ( 3.1 ) | 1.95 ( 0.28 ) |
| **Fall*** | 45 | | 1.5 ( 1.12 - 2.01 ) | 1.49 ( 7.41 ) | 1.49 ( 1.17 ) | 0.58 ( -1.09 ) |
| **Fatigue** | 43 | | 0.61 ( 0.45 - 0.83 ) | 0.62 ( 10.38 ) | 0.62 ( 0.48 ) | -0.7 ( -2.36 ) |
| **Intentional Overdose*** | 42 | | 7.65 ( 5.65 - 10.36 ) | 7.6 ( 240.69 ) | 7.59 ( 5.89 ) | 2.92 ( 1.26 ) |
| **Hallucination*** | 40 | | 6.05 ( 4.43 - 8.26 ) | 6.02 ( 167.36 ) | 6.01 ( 4.63 ) | 2.59 ( 0.92 ) |
| **Feeling Abnormal*** | 40 | | 1.79 ( 1.31 - 2.44 ) | 1.78 ( 13.84 ) | 1.78 ( 1.38 ) | 0.83 ( -0.83 ) |
| **Delirium*** | 39 | | 12.89 ( 9.4 - 17.66 ) | 12.8 ( 423.94 ) | 12.78 ( 9.82 ) | 3.68 ( 2.01 ) |
| **Cardio-Respiratory Arrest*** | 39 | | 10.07 ( 7.35 - 13.8 ) | 10.01 ( 316.11 ) | 10 ( 7.68 ) | 3.32 ( 1.66 ) |
| **Tachycardia*** | 36 | | 4.51 ( 3.25 - 6.26 ) | 4.49 ( 97.75 ) | 4.49 ( 3.41 ) | 2.17 ( 0.5 ) |
| **Serotonin Syndrome*** | 36 | | 21.57 ( 15.54 - 29.94 ) | 21.43 ( 699.94 ) | 21.39 ( 16.25 ) | 4.42 ( 2.75 ) |
| **Dry Mouth*** | 35 | | 4.89 ( 3.5 - 6.81 ) | 4.86 ( 107.43 ) | 4.86 ( 3.68 ) | 2.28 ( 0.61 ) |
| **Headache** | 35 | | 0.61 ( 0.44 - 0.86 ) | 0.62 ( 8.48 ) | 0.62 ( 0.47 ) | -0.7 ( -2.37 ) |
| **Heart Rate Increased*** | 35 | | 3.93 ( 2.82 - 5.48 ) | 3.91 ( 75.98 ) | 3.91 ( 2.96 ) | 1.97 ( 0.3 ) |
| **Nausea** | 34 | | 0.48 ( 0.34 - 0.67 ) | 0.48 ( 19.41 ) | 0.48 ( 0.36 ) | -1.06 ( -2.73 ) |
| **Insomnia** | 34 | | 1.39 ( 0.99 - 1.95 ) | 1.39 ( 3.74 ) | 1.39 ( 1.05 ) | 0.48 ( -1.19 ) |
| **Dyspnoea** | 34 | | 0.66 ( 0.47 - 0.93 ) | 0.66 ( 5.8 ) | 0.66 ( 0.5 ) | -0.59 ( -2.26 ) |
| **Hypotension*** | 33 | | 1.82 ( 1.29 - 2.56 ) | 1.81 ( 12.11 ) | 1.81 ( 1.36 ) | 0.86 ( -0.81 ) |
| **Vomiting** | 32 | | 0.76 ( 0.54 - 1.08 ) | 0.77 ( 2.31 ) | 0.77 ( 0.57 ) | -0.38 ( -2.05 ) |
| **Muscle Spasms*** | 32 | | 1.92 ( 1.35 - 2.71 ) | 1.91 ( 13.94 ) | 1.91 ( 1.43 ) | 0.93 ( -0.73 ) |

Asterisks (*) indicate statistically significant signals. Abbreviations: ROR, reporting odds ratio; PRR, proportional reporting ratio; EBGM, empirical Bayesian geometric mean; EBGM05, the lower limit of the 95% confidence interval of EBGM; IC, information component; IC025, the lower limit of the 95% confidence interval of the IC.
